# Characterization of replication and conjugation of plasmid pWTY27 from a widely distributed *Streptomyces* species

**DOI:** 10.1186/1471-2180-12-253

**Published:** 2012-11-07

**Authors:** Tao Wang, Zhenhua Chen, Qiuxiang Cheng, Min Zhou, Xinli Tian, Pengfei Xie, Li Zhong, Meijuan Shen, Zhongjun Qin

**Affiliations:** 1 Key laboratory of Synthetic Biology, Shanghai Institute of Plant Physiology and Ecology, Shanghai Institutes for Biological Sciences, the Chinese Academy of Sciences, 300 Fenglin Road, Shanghai, 200032, China

**Keywords:** Streptomyces, Plasmid, Replication, Conjugation

## Abstract

**Background:**

*Streptomyces* species are widely distributed in natural habitats, such as soils, lakes, plants and some extreme environments. Replication loci of several *Streptomyces* theta-type plasmids have been reported, but are not characterized in details. Conjugation loci of some *Streptomyces* rolling-circle-type plasmids are identified and mechanism of conjugal transferring are described.

**Results:**

We report the detection of a widely distributed *Streptomyces* strain Y27 and its indigenous plasmid pWTY27 from fourteen plants and four soil samples cross China by both culturing and nonculturing methods. The complete nucleotide sequence of pWTY27 consisted of 14,288 bp. A basic locus for plasmid replication comprised *repAB* genes and an adjacent iteron sequence, to a long inverted-repeat (ca. 105 bp) of which the RepA protein bound specifically *in vitro*, suggesting that RepA may recognize a second structure (e.g. a long stem-loop) of the iteron DNA. A plasmid containing the locus propagated in linear mode when the telomeres of a linear plasmid were attached, indicating a bi-directional replication mode for pWTY27. As for rolling-circle plasmids, a single *traA* gene and a *clt* sequence (covering 16 bp within *traA* and its adjacent 159 bp) on pWTY27 were required for plasmid transfer. TraA recognized and bound specifically to the two regions of the *clt* sequence, one containing all the four DC1 of 7 bp (TGACACC) and one DC2 (CCCGCCC) and most of IC1, and another covering two DC2 and part of IC1, suggesting formation of a high-ordered DNA-protein complex.

**Conclusions:**

This work (i) isolates a widespread *Streptomyces* strain Y27 and sequences its indigenous theta-type plasmid pWTY27; (ii) identifies the replication and conjugation loci of pWTY27 and; (iii) characterizes the binding sequences of the RepA and TraA proteins.

## Background

*Streptomyces* species are widely distributed in natural habitats, such as soils, lakes, plants and some extreme environments [1,2]. They are Gram-positive, mycelial bacteria with high G+C content (often >70%) in their DNA [3]. More than 6000 antibiotics and pharmacologically active metabolites (e.g. antiparasitic and antitumor agents, immuno-suppressants etc.) have been discovered in *Streptomyces* species [4]. *Streptomyces* species usually harbor conjugative plasmids [5]. Modes of plasmid replication in *Streptomyces* include rolling-circle (RC) (e.g. pIJ101, pJV1, pSG5, pSN22, pSVH1, pSB24.2, pSY10 and pSNA1) [6], and uni-directional or bi-directional theta types (e.g. SCP2, pFP11 and pFP1) [7,8]. Some plasmids (e.g. SLP1 and pSAM2) replicate in chromosomally-integrating/autonomous forms [9-11]. *Streptomyces* RC plasmids are usually small (8–13 kb), while theta-type plasmids are larger (31–120 kb).

Replication loci of *Streptomyces* RC plasmids comprise a single *rep* gene, a *dso* (double-strand origin) for initiation and termination of replication, and an *sso* (single-strand origin) for conversion of the lagging strand into a double-stranded molecule [12]. The replication locus of the theta-type SCP2 comprises *repI* and *repII* genes and an adjacent non-coding sequence to which RepI protein binds [7,13]. pFP1 and pFP11 contain basic replication loci of *rep* and iteron types (direct repeats and/or inverted repeats), to which Rep proteins bind [8].

Conjugal transfer of *Streptomyces* RC plasmid (e.g. pIJ101) needs a *tra* gene along with a *clt* (*cis*-acting locus of transfer) site [14]. *Streptomyces tra* genes encode a DNA translocase resembling the chromosomal DNA translocase FtsK of *E. coli* or SpoIIIE of *B. subtilis*[3], with double-stranded DNA probably entering the recipient [15]. The TraB of pSVH1 binds to the *clt* sequence as multimers on the mobilized plasmid and translocates unprocessed DNA at the hyphal tip to a recipient cell [16]. Conjugal transfer of *Streptomyces* theta-type plasmids (e.g. SCP2 and pZL12) requires a major *tra* gene and two adjacent genes [17,18].

In contrast to most bacteria, *Streptomyces* species often harbor linear plasmids [19,20]. Unlike the terminal protein-capped linear replicons of adenoviruses that replicate by a mechanism of strand displacement [21], *Streptomyces* linear plasmids start replication from a centrally located *ori* locus [22] and replication proceeds bi-directionally toward the telomeres [23]. At least some *Streptomyces* linear plasmids (e.g. pSCL1) can propagate in circular mode when the telomeres are deleted [22], while some theta-type circular plasmids (e.g. SCP2 and pFP11) can also propagate in linear mode when the telomeres from a linear plasmid are attached [8].

## Results

### Identification of a widely distributed *Streptomyces* species Y27 and its indigenous plasmid pWTY27 among endophytic *Streptomyces* strains

During the course of investigating naturally circular plasmids, we detected 27 plasmids among ~300 newly isolated actinomycete strains from plant samples of Gingko, Taxus and *Artemisia annua L* in China. Interestingly, 14 of them (Table 1) displayed similar sizes of ca.14-kb DNA bands on agarose gel. These plasmids were digested with *Nco*I and all showed five bands (~8, 2.2, 1.7, 1.3 and 1 kb) on gel electrophoresis (Additional file 1: Figure S1), suggesting that they were an identical plasmid (designated pWTY27).

**Table 1:** Strains and plasmids used in this study

**Strain and plasmid**	**Genotype or description**	**Source or reference**
Strains		
*Streptomyces* strains (Y27, Y32, Y33, Y34, Y41, Y42 and G2-1)	Isolated from Gingko harboring pWTY27	This work
*Streptomyces* strains(W15, W24, W37 and W41)	Isolated from *Artemisia annua L* harboring pWTY27	This work
*Streptomyces* strains (Z20, Z54 and Z70)	Isolated from Taxus harboring pWTY27	This work
*S. lividans* ZX7	*pro-2 str-6 rec-46 dnd* SLP2^-^ SLP3^-^	34
*S. coelicolor* A3(2)	SCP1 SCP2	35
*Escherichia coli* DH5α	*F- deoR recA1 endA1 hsdR17(rk- mk+) phoA supE44 λ- thi-1 gyrA96 relA1*	Invitrogen
*E. coli* BL21 (DE3)	*hsdSB(rB- mB-) λ(DE3 [lacI lacUV5-T7 gene 1 ind1 sam7 nin5])*	Novagen
*E. coli* ET12567 (pUZ8002)	*dam dcm hsdM cm kan*	35
Plasmids		
pSP72	*amp colEI ori*	Invitrogen
pIJ702	*tsr melC pIJ101 ori*	39
pYQ1	A 14-kb *Sac*I-fragment cloned in pSP72	This work
pQC156	A 2.6-kb *Bcl*I-fragment of *melC/tsr* cloned in pSP72 (*Bgl*II)	26
pZR131	Two 381-bp telomeres *tsr melC amp colEI ori*	8
pWT177	A 3.8-kb fragment (100-3941 bp) cloned in pZR131 (*EcoR*I)	This work
pSET152	*amp apr oriT int(phiC31)*	36
pWT181	pSET152 derivative, *amp tsr melC cos oriT int(phiC31)*	This work
pET28b	*kan*	Novagen
pWT111	A 1.6-kb fragment (574-2253 bp of pWTY27) cloned in pET28b (*Eco*RI*+Hin*dIII)	This work
pWT371	A 1.7-kb fragment (8124-9836 bp of pWTY27) cloned in pET28b (*Nhe*I*+Hin*dIII)	This work
pFX144	A 1.3-kb fragment (37-1328 bp of pIJ773 containing *oriT/apr*) cloned in pSP72 (*Xba*I)	This work
pWT26	A 1.3-kb fragment (13-1369 bp of pFX144 containing *oriT/apr*) cloned in pYQ1(EcoRV)	This work
pWT24	A 5.4-kb fragment (13942-14288/1-5114 bp of pWTY27) cloned in pFX144 (*Ssp*I *+ Sac*I)	This work
pWT147	A 3.8-kb fragment (100-3941 bp) cloned in pFX144 (*Xba*I)	This work
pWT219	A 3.2-kb fragment (321-3506 bp) cloned in pFX144 (*Xba*I)	This work
pWT217	A 1.9-kb fragment (321-2267 bp) cloned in pFX144 (*Xba*I)	This work
pWT222	A 2.9-kb fragment (621-3506 bp) cloned in pFX144 (*Xba*I)	This work
pWT223	A 0.3-kb fragment (321-620 bp) containing iteron cloned in pWT222 (*Bam*HI)	This work
pWT241	A 0.15-kb fragment (382-530 bp) containing iteron cloned in pWT224 (*Bam*HI)	This work
pWT34	A 95-bp fragment (1073-1167 bp) deleted from pWT24	This work
pWT33	A 259-bp fragment (2433-2691 bp) deleted from pWT24	This work
pWT203	A 6-kb fragment containing the *rep/rlrA/rorA* of pSLA2 cloned in pFX144 (*Pvu*II)	This work
pWT208	A 3.2-kb fragment (6757-9977 bp) cloned in pWT203 (*Ssp*I)	This work
pWT207	A 1.5-kb fragment (6757-8270 bp) cloned in pWT203 (*Ssp*I)	This work
pWT210	A 2.2-kb fragment (7734-9977 bp) cloned in pWT203 (*Ssp*I)	This work
pWT225	A 2.2-kb fragment (7734-9893 bp) cloned in	This work
pWT224	pWT203 (*Ssp*I)	This work
	A 2.1-kb fragment (7734-9818 bp) cloned in pWT203 (*Ssp*I)	
pWT242	A 175-bp fragment (9803-9977 bp) cloned in	This work
	pWT224 (*Ssp*I)	
pWT262	A 46-bp fragment (9803-9848 bp) cloned in	This work
	pWT224 (*Ssp*I)	
pWT231	A 87-bp fragment (9803-9889 bp) cloned in	This work
	pWT224 (*Ssp*I)	
pWT229	A 100-bp fragment (9803-9902 bp) cloned in	This work
	pWT224 (*Ssp*I)	
pWT239	A 128-bp fragment (9803-9930 bp) cloned in	This work
	pWT224 (*Ssp*I)	
pWT238	A 150-bp fragment (9803-9952 bp) cloned in	This work
	pWT224 (*Ssp*I)	
pWT251	A 134-bp fragment (9844-9977 bp) cloned in	This work
	pWT224 (*Ssp*I)	
pWT259	A 165-bp fragment (9813-9977 bp) cloned in	This work
	pWT224 (*Ssp*I)	
pWT265	A 159-bp fragment (9819-9977 bp) cloned in	This work
	pWT224 (*Ssp*I)	

The 16S rRNA genes of the 14 strains were PCR-amplified and all showed the same sequence, resembling those of *Streptomyces* species (e.g. *S. albidoflavus*, *S. globisporus* and *S. coelicolor*, identity 99%). The chromosomal *oriC* regions of these strains were also PCR-amplified with primers from the conserved *dnaA* and *dnaN* genes and all these *oriC* sequences were identical. As shown in Additional file 2: Figure S2, its 1136-bp non-coding sequence was predicted to contain 25 DnaA binding-boxes (including nine forward and sixteen reverse) of 9 bp ([T/C][T/C][G/A]TCCAC[A/C]), resembling that of typical *Streptomyces* (e.g. 17 DnaA boxes of 9 bp [TTGTCCACA] for *S. lividans*) [24]. The genomic DNA of these strains was digested with *Ssp*I and electrophoresed in pulsed-field gel. As shown in Additional file 3: Figure S3, genomic bands of these strains were identical. These results suggested that the 14 strains were identical (designated *Streptomyces* sp. Y27).

### Sequencing and analysis of pWTY27

The unique *Sac*I-treated pWTY27 was cloned in an *E. coli* plasmid pSP72 for shotgun cloning and sequencing (see Methods). The complete nucleotide sequence of pWTY27 consisted of 14,288 bp with 71.8% GC content, resembling that of a typical *Streptomyces* genome (e.g. 72.1% for *S*. *coelicolor*) [25]. Fifteen open reading frames (ORFs) were predicted by “FramePlot 4.0beta” (Additional file 4: Figure S4); seven of them resembled genes of characterized function, while eight were hypothetical or unknown genes. These ORFs were grouped into two large presumed transcriptional units (*pWTY27.5–4c*, *pWTY27.5–14*; Additional file 5: Table S1). Interestingly, five ORFs of pWTY27.2c resembled these of of pSG2 of *S. ghanaensis* (DNA polymerase, SpdB2, TraA, TraB and resolvase). pWTY27.9 containing a domain (from 246 to 464 amino acids) for DNA segregation ATPase FtsK/SpoIIIE resembled a major conjugation Tra protein of *Streptomyces* plasmid pJV1 (NP_044357). Like other *Streptomyces* plasmids (e.g. SLP1 and SCP2), pWTY27 encodes genes showing similarity to transcriptional regulator *kor* (kill-override), *spd* (plasmid spreading) and *int* (integrase) genes. Unexpectedly, pWTY27.11 resembled a chromosomally encoded phage head capsid in *Nocardia farcinica* IFM 10152, suggesting the occurrence of a horizontal transfer event between plasmid and phage.

### Characterization of replication of pWTY27

**Figure 1:**
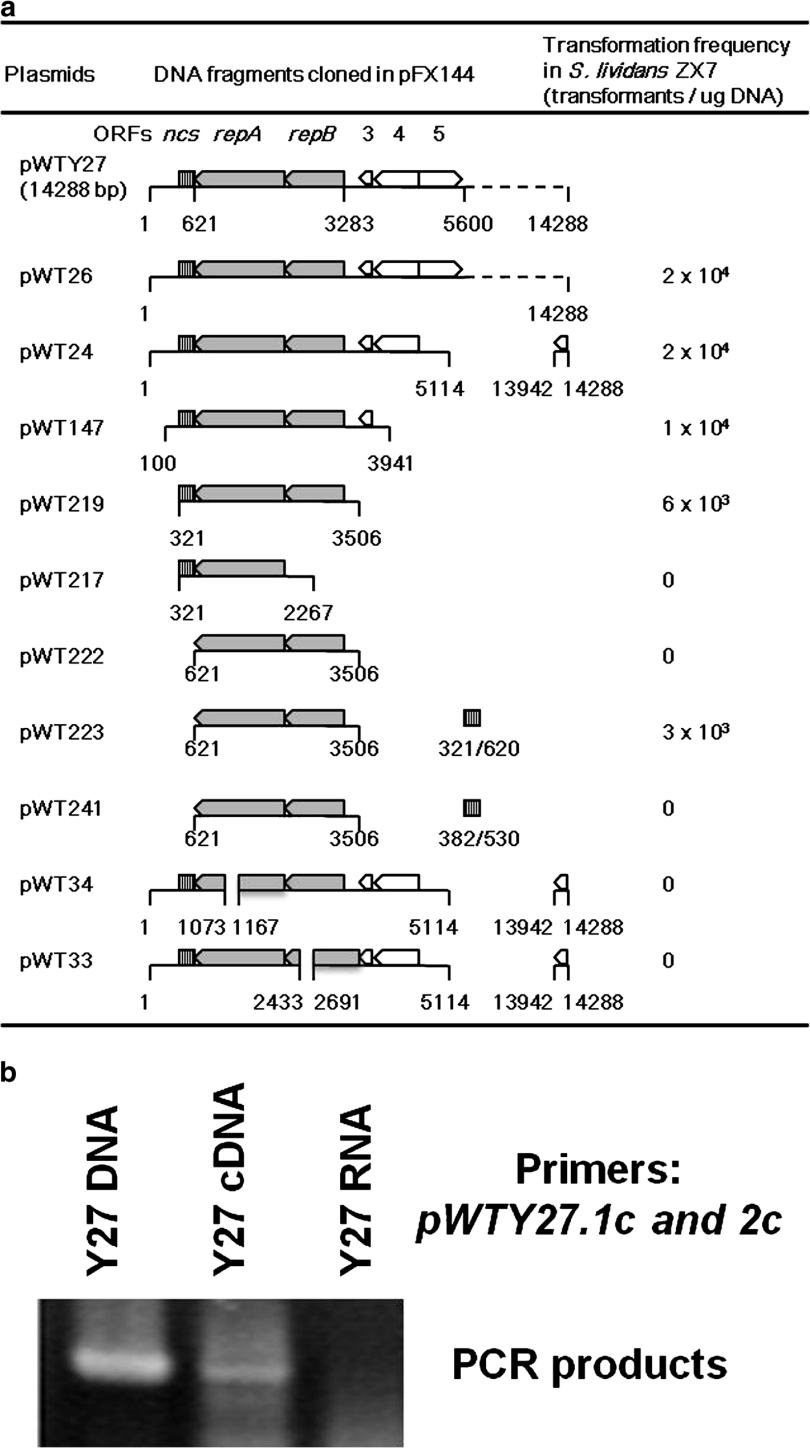
**Identification of a pWTY27 locus required for replication in*Streptomyces lividans.*** (**a**). Identification of a replication locus. Plasmids were constructed in *E. coli* (see Methods and Table 1), and introduced by transformation into *S. lividans* ZX7. Positions of these cloned fragments on pWTY27 and transformation frequencies are shown. The *ncs* is indicated by striped boxes, relevant genes by open arrowheads and the two replication genes by filled arrowheads. (**b**). RT-PCR of a transcript overlapping the consecutive replication genes. RNA of strain Y27 was isolated and reverse-transcribed into cDNA. The cDNA, RNA and Y27 genomic DNA were used as templates for PCR amplification and their products were electrophoresed in 1.5% agarose gel at 20 V/cm for 1 h.

To identify a locus for plasmid replication, various pWTY27 fragments were sub-cloned into an *E. coli* plasmid pFX144 containing a *Streptomyces* apramycin resistance marker and were introduced by transformation into *S. lividans* ZX7. As shown in Figure 1a, plasmids (e.g. pWT24, 26, 147 and 219) containing *pWTY27.1c*, *2c* and a 300-bp non-coding sequence (321–620 bp, *ncs*) could replicate in *S. lividans* ZX7, but deletion of *pWTY27.2c* (i.e. pWT217 and pWT33) or *pWTY27.1c* (pWT34) or the *ncs* (pWT222) abolished propagation in *S. lividans* ZX7. Adding the 300-bp *ncs* (pWT223), but not a 149-bp *ncs* (382–530, pWT241), to pWT222 restored its replication activity. Co-transcription of *pWTY27.1c* and *2c* was confirmed by PCR amplification of their co-transcribed RNA products into cDNA (Figure 1b). These results indicated that a basic locus for pWTY27 replication was *pWTY27.1c* (designated *repA*), *pWTY27.2c* (*repB*) and a 300-bp (from 321 to 620 bp) *ncs*.

pWT26 was introduced by conjugation from *E. coli* ET12567 (pUZ8002) into 10 randomly-selected endophytic *Streptomyces* strains (different 16S rRNA sequences, e.g. Y22, Y45, Y19, Y24, Y8, Y51, Y10, Y31, Y72 and Y3), and apramycin resistant transconjugants were obtained from eight of them, indicating a wide host range for this plasmid.

### RepA protein binds specifically to intact IR2 of the iteron sequence *in vitro*

**Figure 2:**
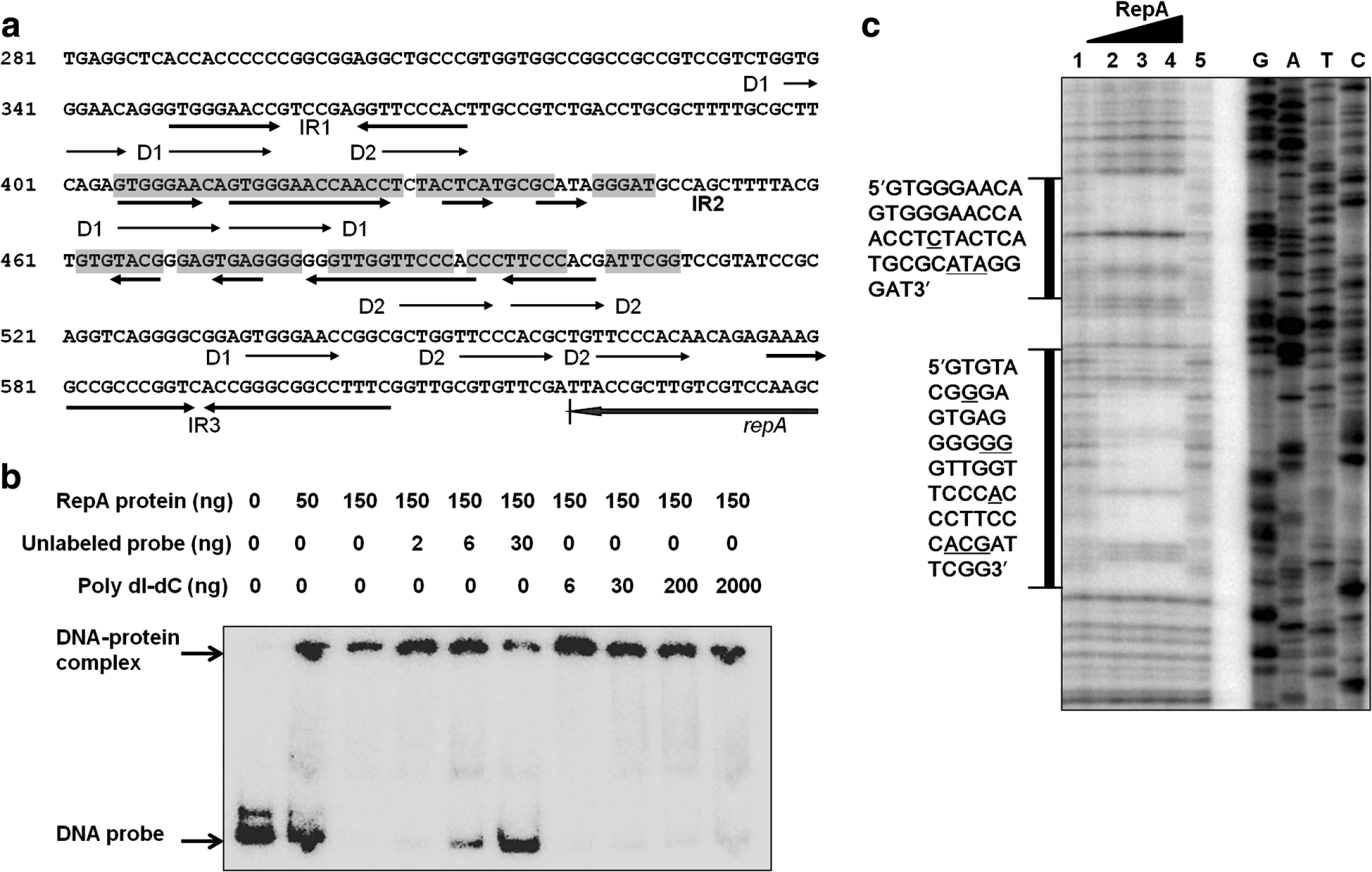
**Characterization of the binding reaction of Rep1A protein with iteron DNA by EMSA and footprinting.** (**a**). Iteron of pWTY27. Possible iteron sequences from 338 to 606 bp on pWTY27 and AT-rich regions are shown. DR: direct repeat; IR: inverted repeat. The RepA binding sequences determined by DNA footprinting are boxed. The binding sequences of RepA protein are indicated by shading. (**b**). Detection of the binding activity of RepA protein with the iteron by EMSA. The DNA probe for each lane was 2 ng and the unlabeled probe was also used as specific competitor. The DNA-protein complex is indicated. (**c**). Determination of the binding sequence by DNA footprinting. The γ[^32^p]ATP-radiolabelled primer was sequenced and electrophoresed (lanes G, A, T and C) as a control. The amounts of RepA protein used in lanes 1–5 were 0.17, 0.43, 0.85, 2.6 and 0 μg, respectively. Two sequences protected by RepA from digestion with DNaseI are shown and the RepA unbound sequences are underlined.

The pWTY27 RepB was predicted to be a DNA primase/polymerase and RepA a hypothetical protein. The 300-bp *ncs* was predicted as an iteron containing five direct repeats of 8 bp (DR1, GTGGGAAC), five direct repeats of 7 bp (DR2, TTCCCAC) and three pairs of inverted repeats (IR1–IR3, Figure 2a). To see if there was an interaction between the RepA protein and this iteron sequence, electrophoretic mobility shift assays for DNA-protein complex formation were employed. The 6His-tagged RepA protein was incubated with a [γ-^32^P]ATP-labeled iteron DNA, and then electrophoresed and autoradiographed. As shown in Figure 2b, the “shifted” DNA bands were visualized by adding RepA protein, indicating that the RepA protein could bind to the DNA probe to form a DNA-protein complex. Formation of this complex was inhibited by adding a 15-fold excess of unlabeled probe but was not affected by adding even a 1000-fold excess of polydIdC DNA as a non-specific competitor, indicating that the binding reaction of the RepA protein with iteron DNA was highly specific.

To precisely determine the binding sequence of the RepA protein and iteron DNA, a “footprinting” assay was employed. As shown in Figure 2c, two sequences (405–447 bp and 462–509 bp) protected from digestion with DNaseI were visualized on adding RepA protein. These sequences (405–509 bp) covered intact IR2 (overlapping with some DR1 and DR2) of the iteron (Figure 2a).

### A plasmid containing the replication locus of pWTY27 propagates in linear mode when the telomeres of a linear plasmid are attached

**Figure 3:**
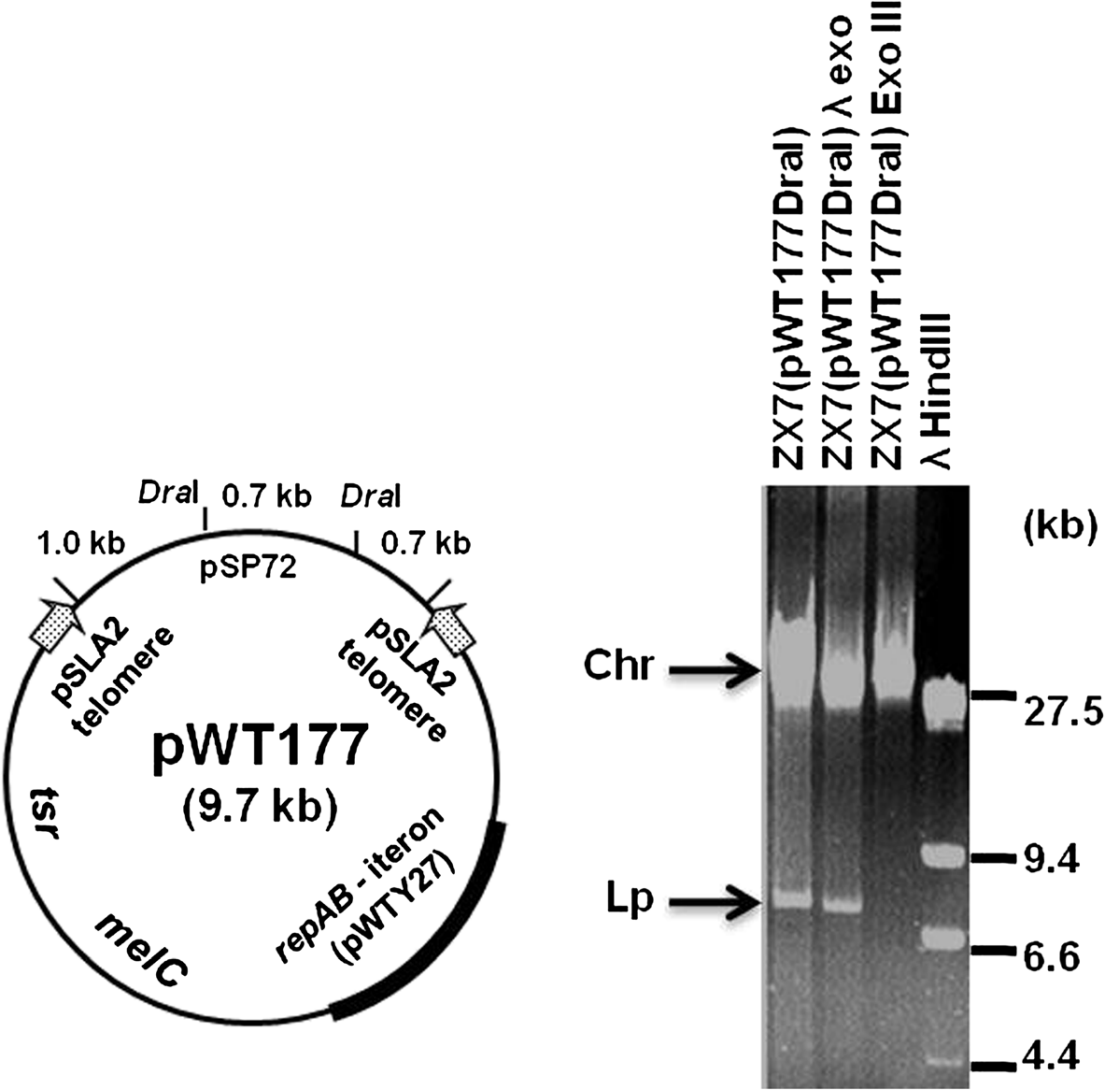
**A plasmid containing the pWTY27 replication locus and pSLA2 telomeres propagated in linear mode in*Streptomyces.*** Aliquots of genomic DNA were treated with *E. coli* exonuclease III and bacteriophage λ exonuclease and electrophoresed in 0.7% agarose gel at 1.3 V/cm for 12 h. Chromosomal (Chr) and linear plasmid (Lp) bands are indicated.

The replication locus of pWTY27 comprised *rep* and an iteron, resembling those of bi-directionally replicating *Streptomyces* plasmids (e.g. pFP11) [8]. To see if pWTY27 could also replicate in linear mode when the telomeres of a linear plasmid were attached, we constructed pWT177 (Figure 3), containing the replication locus of pWTY27, and two 381-bp functional telomeres of linear plasmid pSLA2 [26]. *Dra*I-linearized pWT177 DNA from *E. coli* was introduced by transformation into *S. lividans* ZX7. Transformants were obtained at a frequency of 5 × 10^3^/μg DNA. Genomic DNA was isolated, and a ~7.3-kb plasmid DNA band was detected on an agarose gel. As shown in Figure 3, this band was resistant to treatment by *λ* exonuclease but sensitive to *E. coli* exonuclease III, suggesting that it was a double-stranded linear DNA with free 3^′^ but blocked 5^′^ ends. 

### Identification of a *tra* gene and its adjacent essential sequence for plasmid transfer

**Figure 4:**
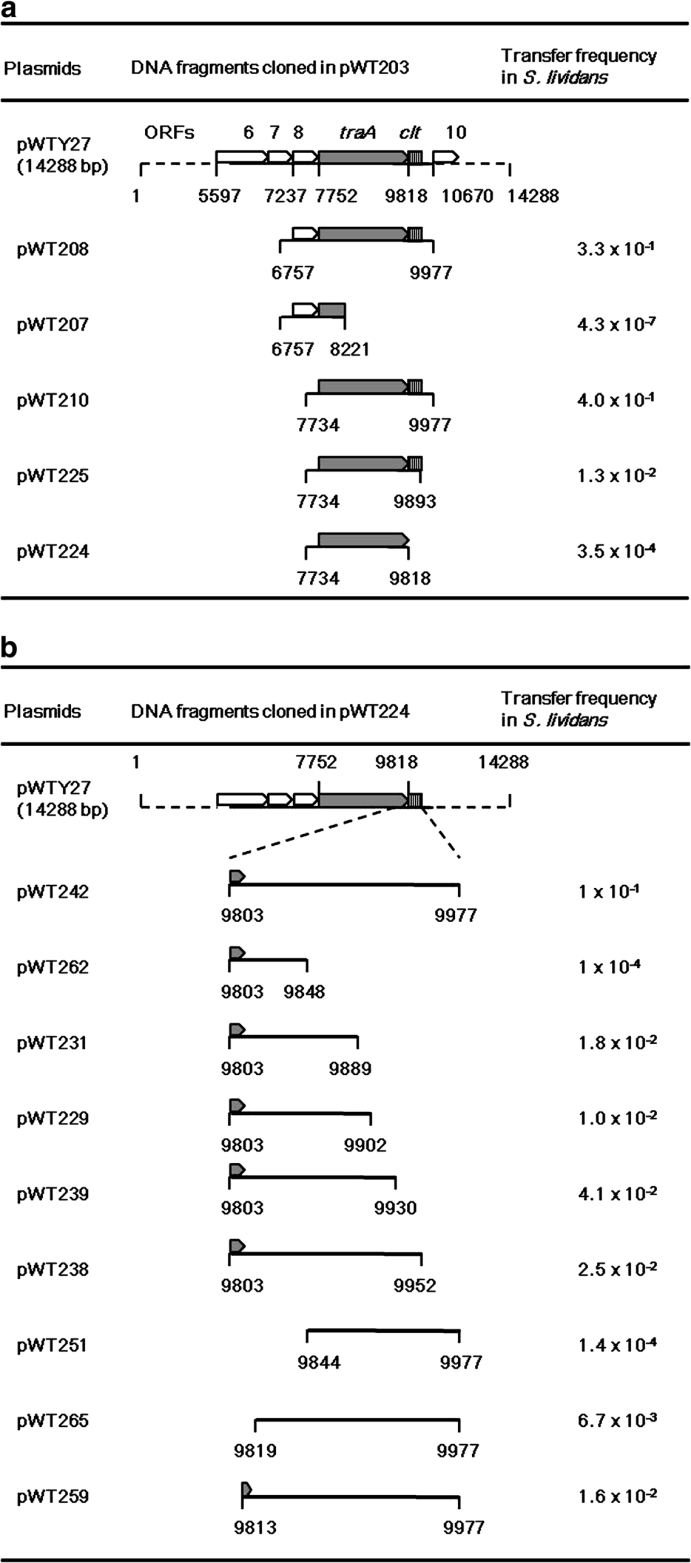
**Identification of a pWTY27 locus for conjugal transfer in*Streptomycescxx*(a) and (b).** Transfer frequencies of the plasmids in *Streptomyces lividans* are shown. Relevant genes are indicated by open arrowheads while *tra* is indicated by an arrowhead, and a possible *clt* by striped boxes.

pWTY27.9 resembled the major conjugation protein Tra of *Streptomyces* plasmid pJV1 [27]. As shown in Figure 4a, plasmids (e.g. pWT208 and pWT210) containing *pWTY27.9* and its adjacent 159-bp sequence (9819–9977) could transfer at high frequencies. Deletion of *pWTY27.9* (pWT207) abolished transfer of the plasmid. Complete (pWT224) or partial deletion (pWT225) of the 159-bp sequence decreased transfer frequencies ca. 1000- and 10-fold, respectively. Thus, a basic locus for pWTY27 transfer comprised *pWTY27.9* (designated *traA*) and its adjacent ~159-bp sequence. 

To precisely determine the essential segment of the short sequence for plasmid transfer, various fragments were PCR-amplified and then cloned into pWT224 containing intact *traA* but not the 159-bp sequence. As shown in Figure 4b, a plasmid (pWT242) containing a 175-bp fragment (a 16-bp sequence within *traA* and the 159-bp non-coding sequence, *cis*-acting-locus of transfer, designated *clt*) could transfer at a high frequency. Deletions of 10 bp within *traA* (pWT259) decreased transfer frequency ca. 1000-fold. Deletions of 88 bp (pWT231) and 129 bp (pWT262) of the *clt* decreased transfer frequencies ca. 10- and 1000-fold, respectively. These results suggested that the essential region for plasmid transfer was ca. 87 bp covering 16 bp within *traA* and its adjacent 71 bp (9803–9889), while the 88 bp (9890–9977) next to it also played a role in plasmid transfer.

### TraA protein binds specifically to the *clt* sequence *in vitro*

**Figure 5:**
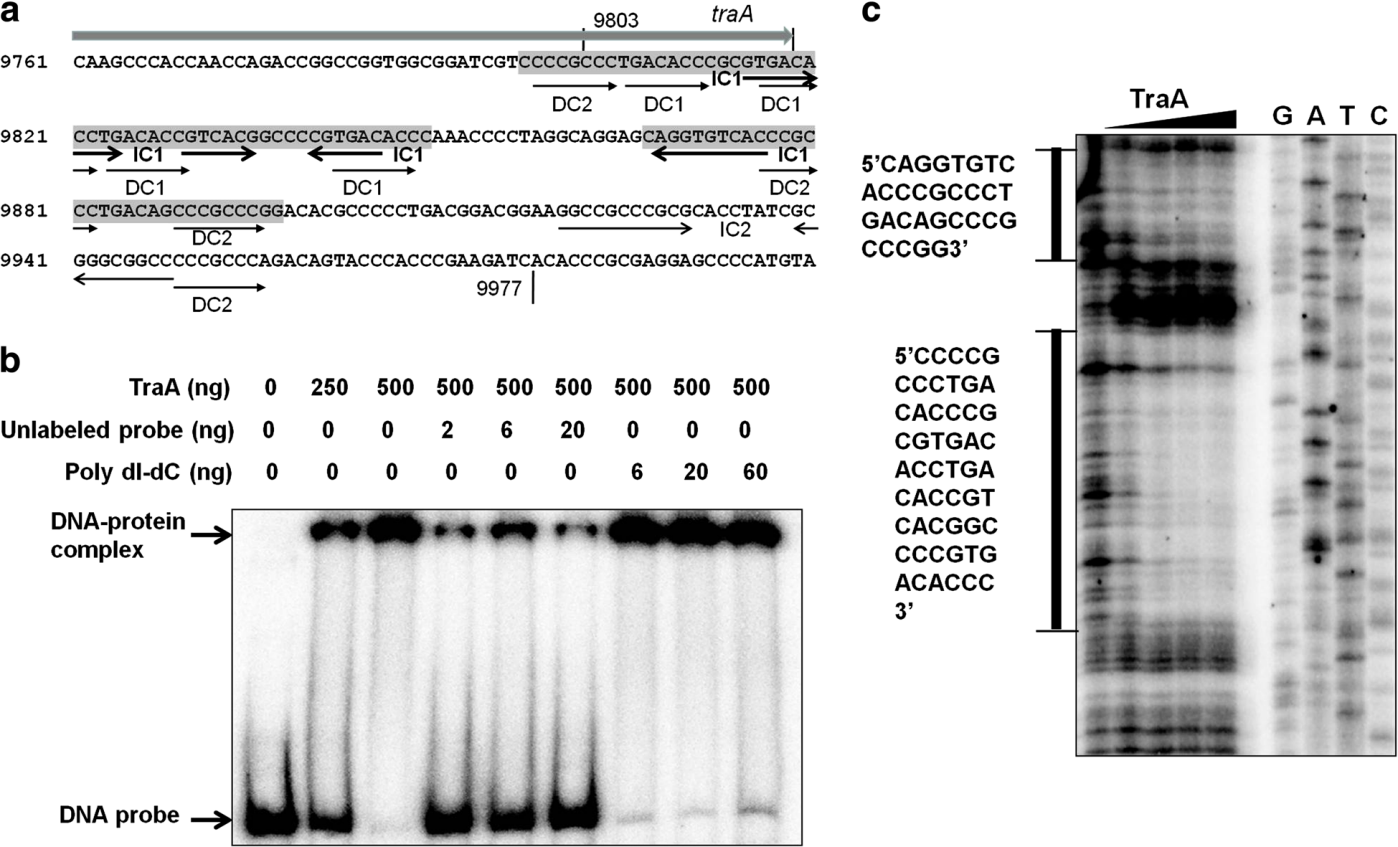
**Characterization of the binding reaction of TraA protein with*clt*DNA by EMSA and footprinting.** (**a**). Characteristics of a *clt* sequence on pWTY27 for plasmid transfer. Possible DC (direct repeat) and IC (inverted repeat) sequences are shown. (**b**) as **Figure**2**(b).** (**c**) as **Figure**2**(c).** The amounts of TraA protein used in lanes 1–5 were 0, 0.6, 1.4, 2.8 and 4.2 μg, respectively. Two sequences protected by TraA from digestion with DNaseI are shown.

Two trans-membrane domains (68–90 and 102–124 aa) in the 688-aa TraA protein were predicted (http://www.cbs.dtu.dk/services/TMHMM-2.0/). A truncated TraA (125–688 aa) lacking the trans-membrane domains could be expressed in *E. coli* as soluble protein. The 175-bp *clt* sequence (9803–9977) contained four direct repeats (DC1, TGACACC; DC2, CCCGCCC) and two inverted repeats (IC1 and IC2) (Figure 5a). To see if there was an interaction between TraA protein and the *clt* sequence, a “band-shift” assay for DNA-protein complex formation was employed. As shown in Figure 5b, TraA protein could bind to the DNA probe to form a DNA-protein complex. Formation of this complex was inhibited by adding 1–10 fold excess of unlabeled probe but was not affected by adding a 30-fold (even 1000-fold, data not shown) excess of polydIdC DNA as a non-specific competitor, indicating that the binding reaction of the TraA protein with the *clt* DNA was highly specific.

A “footprinting” assay was employed to precisely determine the binding sequence of TraA protein and *clt* DNA. As shown in Figure 5c, two sequences (9797–9849 bp and 9867–9897 bp) protected from digestion with DNase I were visualized on adding TraA protein. One sequence (9797–9849 bp) covered all the four DC1 and one DC2 and most of IC1, and another (9867–9897 bp) covered two DC2 and part of IC1 of the *clt* (Figure 5a). The pWTY27 sequence contained four DR1 within the *clt* while carrying fifteen DC2 distributing randomly in the plasmid, suggesting an essential role of the DC1 for plasmid transfer.

### Detection of a variety of *repA* of pWTY27 and *oriC* sequences of Y27 among soil samples

**Figure 6:**
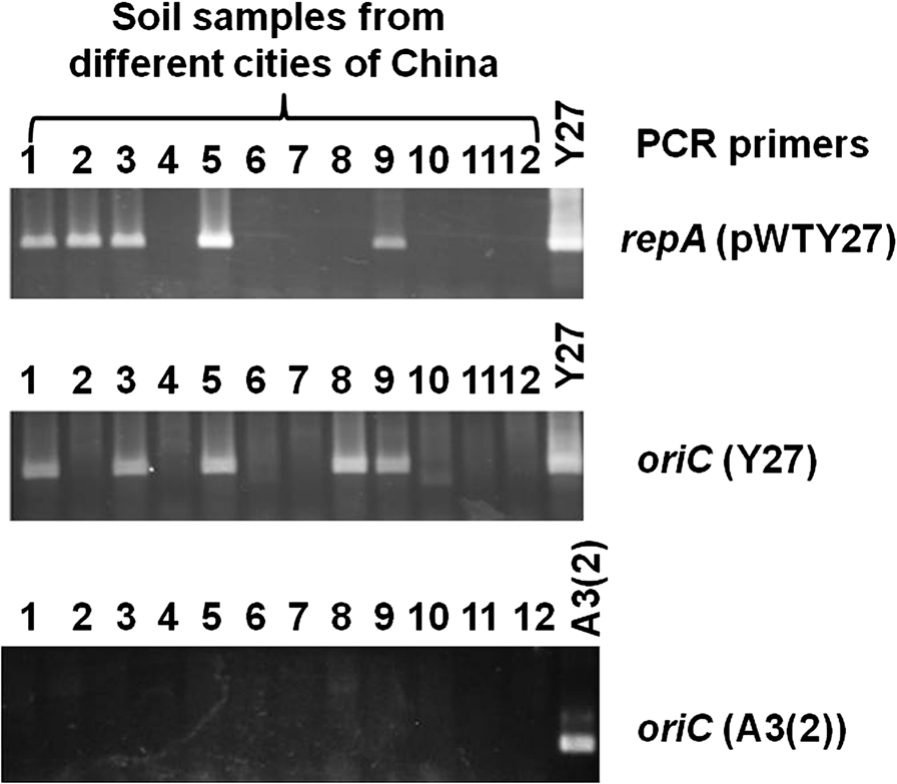
**PCR amplifications of possible pWTY27*repA*and*oriC*from the genomic DNA of soil samples.** Twelve soil samples (lanes 1–12) were collected, soil genomic DNA was isolated and nested PCR amplifications with primers of the pWTY27 *repA*, Y27 and A3(2) *oriC* were performed (Methods).

By detecting the indigenous plasmid pWTY27, we have identified a widely distributed *Streptomyces* strain Y27 among plant samples. To see if this species along with the plasmid could also reside in soil, we collected 12 soil samples from 12 cities of nine provinces in China. Soil genomic DNA was isolated and PCR-amplified with primers from the *repA* of pWTY27 and the *oriC* of Y27. As shown in Figure 6, PCR bands were visualized from five samples (1, 2, 3, 5 and 9) for *repA* and also five samples (1, 3, 5, 8 and 9) for *oriC*, while no PCR bands were obtained for the *oriC* of *S. ceolicolor* A3(2) from the twelve soil samples. There was a correlation between the *repA* and the *oriC* in four samples (1, 3, 5 and 9), while *repA*, but not *oriC*, was detected from sample 2, and *oriC*, but not *repA*, from sample 8. These PCR bands were sequenced, showing that the *repA* sequences of samples 1 and 5 were identical to that of pWTY27, while one point mutation (C changed to A at 1878-bp of pWTY27) was found in that of sample 3, one mutation (G to T at 1895 bp) in sample 9, and twelve point mutations in sample 2. The *oriC* sequences of samples 1, 3, 8 and 9 were identical to that of Y27, while there was one point mutation (C to A at 955-bp of the 1433-bp *oriC* sequence) in sample 5. These results indicated that a number of point mutations for the *repA* and *oriC* occurred from these soil samples.

## Discussion

More than 500 species or sub-species in the genus *Streptomyces* have validly been designated and published [28]. However, whether there was some predominant *Streptomces* species in natural habitats was not clear. From six isolates of an endophytic (wheat plant) *Streptomyces* species across South Australia, a 12,855-bp plasmid pEN2701 was identified [29]. Here, we report identification of a 14,288-bp plasmid pWTY27 in an endophytic *Streptomyces* species Y27 from fourteen plant samples of Gingko, Taxus and *Artemisia annua L* across China. By integrating the egfp gene (encoding green fluorescence protein) in the Y27 chromosome and then infecting leaves of Ginkgo, however, we could not detect Y27 strains growing inside the leaves (T. Wang and Z. Qin, unpublished data). By PCR amplification of soil genomic DNA and sequencing, we found that four out of the 12 soil samples collected from 12 cities in China contained similar *repA* of pWTY27 and *oriC* of Y27. However, the absence of pWTY27-*repA* and YT27-*oriC* in certain soil samples can also be explained by the presence of a PCR-inhibitor (e.g. contamination with humic acids) in the soil samples that gave negative results. The sequence of pWTY27 does not resemble that of pEN2701, and the *oriC* sequence of Y27 is unique in the GenBank database. Thus, we identified a widely distributed *Streptomyces* species along with its indigenous plasmid from some plants and soils cross China by both culturing and nonculturing methods. Existence of a widely distributed species in natural habitats might reflect a versatile capacity to resist stresses.

**Figure 7:**
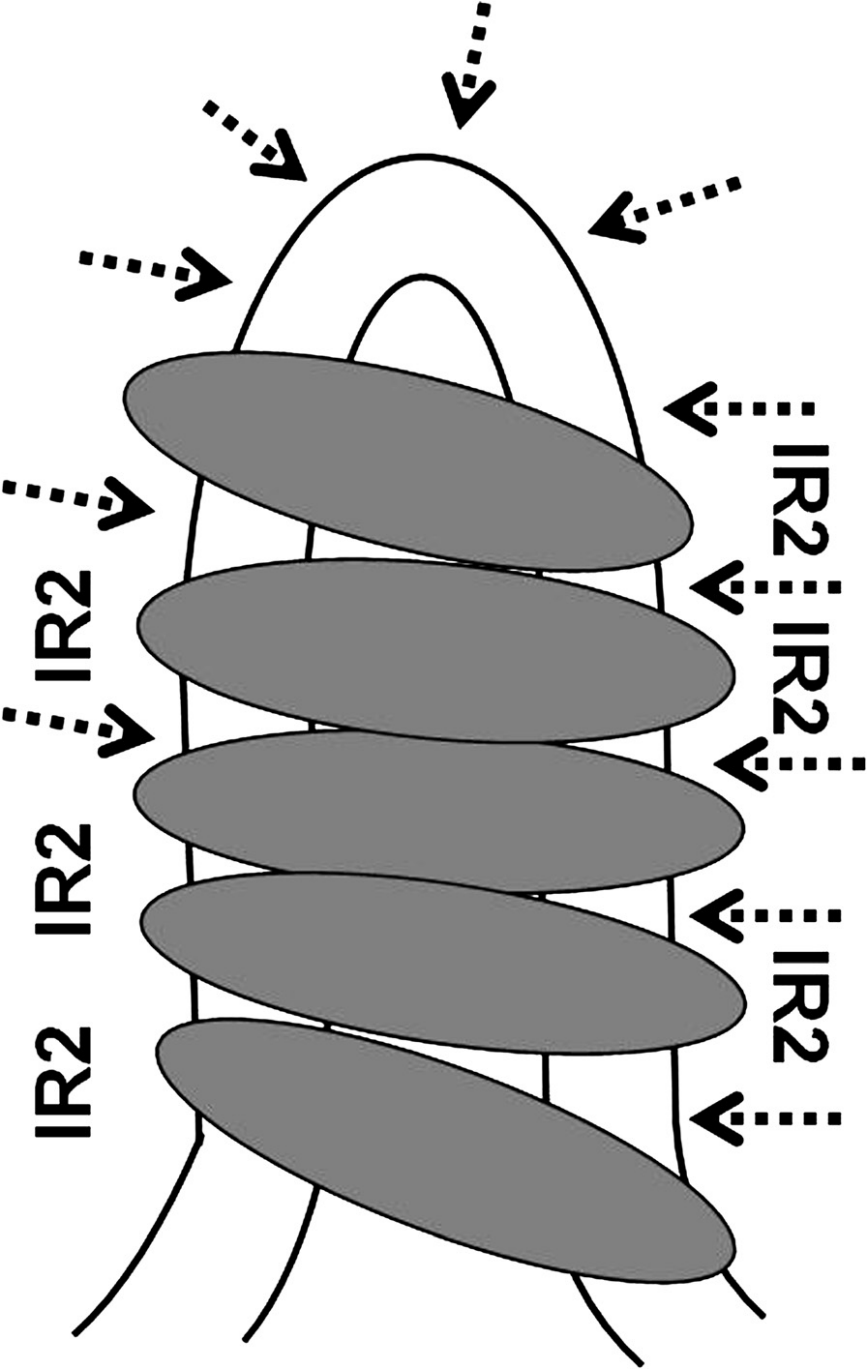
**A model for interaction of the pWTY27 RepA and the iteron.** The replication origin of plasmid pWTY27 contains multiple directed and inverted repeat sequences (DRs and IRs, Figure 2a). The IR2 is a long discontinous inverted-repeat sequence and may fold back itself during initiation of replication. Since there are six unbound sites (see Figure 2a) and RepA is a large protein (522 amino acids), we suggest that five RepA molecules (indicated by filled ovals) may bind to the folding-back IR2 region leaving six unbound sites (indicated by arrowheads).

The basic replication locus of pWTY27 comprises *repAB* genes and an iteron sequence, resembling that of *Streptomyces* theta-type plasmids SCP2 (*repI/repII*) [13], pFP11 and pFP1 (*repA/iteron*) [8]. Given the model of bi-directional replication of *Streptomyces* linear replicons [23], like SCP2 and pFP11 [8], the pWTY2-*rep* locus with artificially attached telomeres from a *Streptomyces* linear plasmid is also able to propagate in linear form, indicating that it replicates in a bi-directional mode. The RepI of SCP2 binds to an upstream sequence of the *repI* gene [7]. The RepA proteins of pFP1 and pFP11 bind specifically to their iterons [8]. The RepA of pWTY27 also binds highly specifically to the iteron *in vitro*, and further DNA “footprinting” showed that the protein binds to intact IR2, which overlaps with some DR1 and DR2, but leaving some spacers, especially the “loop” of the IR2 unprotected from digestion with DNaseI. The long IR2 sequence may fold back to form hairpin structure. In fact, DR2 (GTGGGAAC) is almost the complementary sequence of DR1 (TTCCCAC), which means it is the same repeat but on the opposite strand. These results suggest that RepA may form multimers and recongnize a second structure (e.g. long stem-loop of the IR2) of the iteron DNA (Figure 7). 

Conjugal transfer of *Streptomyces* theta-type plasmids (e.g. SCP2 and pZL12) requires a major *tra* and its adjacent genes [17,18], while that of *Streptomyces* RC-type plasmids (e.g. pIJ101 and pJV1) needs a *tra* gene and a *clt* site [14,30]. The minimal pIJ101 *clt*-locus consists of a sequence ~54 bp in size that includes an essential imperfect inverted repeat and three direct repeats (5 bp, GC/AAAC) sequences and is located close to the *korB* gene [31]. The pJV1 *clt* region contains nine direct repeats (9 bp, CCGCACA[C/G][C/G]) and two pairs of imperfect inverted repeats [30,32]. Like these *Streptomyces* RC-type plasmids, conjugal transfer of the theta-type pWTY27 requires a major *tra* gene and its adjacent sequence. Such a *clt* locus in pWTY27 has a 16-bp sequence within the *traA* gene. The 175-bp *clt* sequence contains four direct repeats of DC1 (7 bp, TGACACC)/DC2 (7 bp, CCCGCCC) and two inverted repeats (IC1 and IC2). Thus, although the *clt* sequences of *Streptomyces* conjugative plasmids are varied, they contain multiple direct repeats and/or inverted repeats.

Reuther *et al*. [16] report that TraB protein of pSVH1 binds to a 50-bp *clt*-like sequence containing a 14-bp direct repeat, producing a protein-DNA complex too large to enter an agarose gel, indicating that multimers of TraB are bound to the DNA. Vogelmann *et al*. [33] show that TraB specifically recognizes repeated 8-bp motifs on pSVH1 mediated by helix α3 of the C-terminal winged-helix-turn-helix domain of the protein, and TraB assembles as a hexameric ring structure with a central 3.1-nm channel and forms pores in lipid bilayers. By removing the N-terminal trans-membrane domain, TraA of pWTY27 can be expressed in *E. coli* as a soluble protein. TraA recognizes and binds specifically to two regions, one (9797–9849 bp) containing all the four DC1 and one DC2 and most part of IC1 and another (9867–9897 bp) covering two DC2 and part of IC1 of the *clt*, suggesting that formation of a high-ordered protein-DNA complex.

## Conclusions

In this work, a widely distributed *Streptomyces* strain Y27 along with its indigenous plasmid pWTY27 from plants and soil samples cross China are identified by both culturing and nonculturing methods. The complete nucleotide sequence of pWTY27 consists of 14,288 bp. A minimal locus for plasmid replication comprises *repAB* genes and an adjacent iteron sequence. RepA protein binds specifically *in vitro* to a long inverted-repeat (i.e. IR2) of the iteron sequence. Plasmid containing the replication locus and two telomeres from *Streptomyces* linear plasmid can propagate in linear mode, indicating a bi-directional replication mode for pWTY27. As for rolling-circle plasmids, a single *traA* gene and a *clt* sequence on pWTY27 are required for plasmid transfer. We find that TraA binds specifically to the two regions of the *clt* sequence, one containing all the four DC1 of 7 bp (TGACACC) and one DC2 (CCCGCCC) and most of IC1, and another covering two DC2 and part of IC1, suggesting formation of a high-ordered DNA-protein complex.

## Methods

### Bacterial strains, plasmids, and general methods

Strains and plasmids used in this study are listed in Table 1. *Streptomyces lividans* ZX7 [34] was the host for plasmid propagation and conjugal transfer. *Streptomyces* culture, isolation of plasmid and genomic DNA, preparation of protoplasts and transformation, and pulsed-field gel electrophoresis followed Kieser *et al*. [35]. Plasmid conjugation from *E. coli* ET12567 (pUZ8002) into *Streptomyces* strains followed Bierman *et al*. [36]. Plasmids pSP72 and pFX144 were used as cloning vectors. *E. coli* strain DH5α was used as cloning host. Plasmid isolation, transformation of *E. coli* and PCR amplification followed Sambrook *et al*. [37].

### Isolation and identification of endophytic *Streptomyces*

Isolation of endophytic actinomycetes from Chinese medicinal herbs followed Cao *et al*. [38]. The plant samples were submerged sequentially in 75% ethanol for 5 min, 0.9% sodium hypochlorite for 10 min, 10% sterile sodium bicarbonate for 10–20 min (10 min for leaf, 20 min for stem) and then washed by sterile water three times. The samples were cut into 1-cm^2^ pieces and were inserted in different media (e.g. TSB [Tryptone Soya Broth powder 30 g, agar 20 g/L] S [glucose 10 g, tryptone 4 g, K_2_HPO_4_·3H_2_O 0.5 g, MgSO_4_·7H_2_O 0.1 g, CaCl_2_·2H_2_O 0.1 g, Ferric citrate reserving solution (1% (w/v) citric acid, 1% (w/v) ferric citrate) 1 ml, trace element solution (H_3_BO_3_1.5 g, MnSO_4_·H_2_O 0.49 g, ZnSO_4_·7H_2_O 0.6 g, CuSO_4_·5H_2_O 0.1 g, (NH_4_)_6_(Mo_7_O_2_)_4_·4H_2_O 0.2 g, CoSO_4_·7H_2_O 0.01 g) 1 ml, agar 20 g/L] and Gause’s synthetic agar [soluble starch 20 g, KNO_3_, 1 g, NaCl 0.5 g, K_2_HPO_4_·3H_2_O 0.5 g, MgSO_4_·7H_2_O 0.5 g, FeSO_4_·7H_2_O 0.01 g, agar 20 g/L]) containing 25 ppm K_2_Cr_2_O_4_, 15 ppm nalidixic acid and 25 ppm nystatin. After incubation at 30°C for four weeks, actinomycete colonies were picked. Actinomycete strains were identified as *Streptomyces* strains by PCR amplification (primers: 5^′^-AGAGTTTGATCCTGGCTCAG-3^′^ and 5^′^-TCAGGCTACCTTGTTACGACTT3^′^) and sequencing of the 16S rRNA genes. The sequence of the 16S rRNA gene of Y27 was deposited in the GenBank under accession number JN207128.1.

### Cloning and sequencing of *Streptomyces* plasmid pWTY27

pWTY27 DNA was digested with restriction endonucleases *Apa*I, *Bam*HI, *Bcl*I, *Bgl*II, *Cla*I, *Eco*RI, *Hin*dIII, *Kpn*I, *Mlu*I, *Nco*I, *Nhe*I, *Pst*I, *Sac*I, *Xba*I and *Xho*I to make a restriction map, and the unique *Sac*I-digested plasmid DNA was cloned into pSP72 to obtain pYQ1. Shotgun cloning and sequencing of pYQ1 were performed on an Applied Biosystems Genetic Analyzer model 377 at the Chinese Human Genome Center in Shanghai. Analysis of *Streptomyces* protein coding regions was performed with “FramePlot 4.0 beta” (http://nocardia.nih.go.jp/fp4/), and ATG or GTG or TTG was used as start codons. Sequence comparisons and protein domain searching were done with software from the National Center for Biotechnology Information (http://www.ncbi.nlm.nih.gov/Blast.cgi). DNA secondary structures (e.g. direct repeats and inverted repeats) were predicted with “DNA folder” (http://mfold.rna.albany.edu/?q=mfold/DNA-Folding-Form) and “Clone manager version 9” (http://www.scied.com/pr_cmpro.htm). The GenBank accession number for the complete nucleotide sequence of pWTY27 is GU226194.2.

### Identification of a locus of pWTY27 for replication in *Streptomyces lividans*

Apramycin resistant transformants in *S*. *lividans* ZX7 were obtained for plasmid pWT24 carrying a 5.4-kb fragment (13942–14288/1–5114 bp of pWTY27). Various segments of the 5.4-kb sequence were PCR amplified and cloned in pFX144 to obtain plasmids pWT147, pWT219, pWT217 and pWT222. Sequences of 95 bp (1073–1167 and 259 bp (2433–2691 of pWT24 were deleted by digesting with *Mlu*I and *Not*I to obtain pWT34 and pWT33, respectively. These pWTY27-derived plasmids were constructed in *E. coli* DH5α and introduced by transformation into *S. lividans* ZX7. To compare transformation frequencies of plasmids in different experiments, we used 0.1 ng DNA (diluted from a concentrated solution) of *Streptomyces* plasmid pIJ702 [39] each time and took 1 × 10^6^ transformants per μg DNA as a control frequency.

### Reverse transcription PCR assay

Strain Y27 was inoculated into tryptone soya broth (TSB, Oxoid) liquid medium, and RNA was isolated following Kieser *et al*. [35]. The RNA samples were treated with DNase I (RNase-free, Takara) to remove possible contaminating DNA and reverse-transcribed into cDNA by using SuperScriptTM III Reverse Transcriptase (Invitrogen). Two primers (5^′^-GTGAATCTTGGGCTCGCCCTTG-3^′^/5^′^- GCCGAGAAGTGCATCCGCAAC-3^′^; the expected size of the PCR product is 302 bp) were used to allow amplification of segments extending from each replication gene into its immediate neighbor. PCR conditions were: template DNA denatured at 95°C for 5 min, then 95°C 30 s, 58°C 30 s, 72°C 30 s, for 30 cycles.

### Electrophoretic mobility shift assay (EMSA)

The *repA* gene (621–2198 bp) of pWTY27 was cloned into the *Eco*RI and *Hin*dIII sites of *E. coli* plasmid pET28b to obtain pWT111, which was then introduced into *E. coli* BL21 (DE3). 1 mM IPTG (isopropyl-β-D-thiogalactopyranoside) was added to a log-phase culture at 16°C for 12 h to induce over-expression of the cloned gene. The 6His-tagged RepA protein was eluted in buffer containing imidazole and was purified to ~90% homogeneity by Ni^2+^ column chromatography following the supplier’s instructions (Qiagen). The 300-bp sequence (321–620) was PCR-amplified and end-labeled with [γ-^32^P]ATP using T4 polynucleotide kinase (New England BioLabs). The DNA-binding reaction was performed at room temperature for 10 min in buffer (20 mM Tris–HCl at pH7.5, 100 mM NaCl, 1 mM ATP and 10% glycerol). PolydIdC DNA was used as non-specific competitor and unlabeled probe as specific competitor. The reaction complexes were separated on a 5% native polyacrylamide gel in 0.5× Tris-borate-EDTA buffer at 120 V for 1 h. Gels were dried and analyzed using the Phosphorimager (Fuji).

Similarly, the truncated *traA* gene (8124–9836 bp) of pWTY27 was cloned in pET28b to yield pWT371. The 6His-tagged TraA protein was purified by Ni^2+^ column chromatography and was incubated with the 175-bp (9803–9977) PCR fragment labeled with [γ-^32^P]ATP at room temperature for 15 min.

### DNA footprinting

The DNase I footprinting assay followed Pan *et al*. [40]. Primer FTr (5^′^-TCGAACACGCAACCGAAAGGCCG3^′^) was end-labeled with [γ-^32^P]ATP using T4 polynucleotide kinase, and then a 300-bp (321– 620) DNA fragment was PCR-amplified with primers ^32^PFTr and FTf (5^′^-CGGCCGCCGTCCGTCTGGTG-3^′^), followed by purification with the Wizard SV Gel and PCR Clean-Up System (Promega). Ca. 40-ng labeled DNA and different amounts (0.17, 0.43, 0.85 and 2.6 μg) of the purified RepA protein were incubated at room temperature for 10 min in a 56-μl binding buffer (20 mM TrisHCl pH 7.5, 100 mM NaCl, 1 mM ATP-Na, 10% glycerol). 1 Unit DNase I (Promega) was added for 1 min and the reaction was stopped by adding 50 μl stop solution (20 mM EGTA, pH 8.0). DNA was extracted with acid phenol/chloroform solution and precipitated with isopropanol and ethanol. Sequencing ladders were prepared with FTr using the SILVER SEQUENCETM DNA Sequencing Reagents (Promega). The digestion products together with the ladders were analyzed in 6% polyacrylamide (adding 7 M urea) gel. Gels were dried and scanned with the Phosphorimager.

Similarly, to determine the binding sequence of TraA protein and *clt* sequence, primer Fcltf (5^′^-CAAGGACTTCATGGACTGGTGCGA-3^′^,) was end-labeled with [γ-^32^P]ATP, and then a 406-bp (9671–10077) DNA fragment was PCR-amplified with primers ^32^PFcltf and Fcltr (5^′^-CGTGCTCGGCCTGCTCCAGGA-3^′^). About 40 ng labeled DNA and different amounts (0.6, 1.4, 2.8 and 4.2 μg) of the purified TraA protein were incubated at room temperature for 15min.

### Identification of a locus for pWTY27 transfer in *Streptomyces lividans*

To identify a locus for plasmid conjugal transfer, various pWTY27 fragments around pWTY27.9 were cloned in *E. coli* plasmids pWT203 which contained the *rep/rlrA/rorA* genes required for replication and stable inheritance of the non-conjugative *Streptomyces* plasmid pSLA2 (31) or pWT224 (carrying intact *traA*). These plasmids were introduced by transformation into *S. lividans* ZX7 to produce donor strains for conjugation. The recipient strain was *S. lividans* ZX7 with a chromosomally integrating plasmid pWT181 containing the *integrase* gene of ΦC31 [41] and selection marker *tsr*. About equal amount (ca.10^8^) of spores of the donor and recipient strains were mixed and incubated at 30°C for 5 days. Spores were harvested, diluted in water and plated equally on Luria-Bertani (LB) medium (thiostrepton, 50 mg/L), LB (apramycin, 50 mg/L) and LB (thiostrepton + apramycin). The frequency of plasmid transfer = 100 × ratio of colonies on LB (thiostrepton + apramycin) to colonies on LB (apramycin).

### Isolation of soil genomic DNA and PCR amplifications of the pWTY27 *repA* and *oriC*

Twelve soil samples from 12 cities in nine provinces (Wuhan, Huanggang and Xianning cities of Hubei, Changde and Hengyang of Hunan, Nanjin of Jiangsu, Linyi of Shandong, Anyan of Henan, Xingtai of Hebei, Guiling of Guangxi, Shanghai, and HongKong) in China were collected. Ca. 0.2-g soil sample and 0.5 g glass beads mixed in 1 ml buffer SLX Mlus were vibrated for 5 min and then were lysed in buffer DS at 90°C for 10 min. Crude genomic DNA was isolated by using the E.Z.N.A^TM^ Soil DNA Kit (Omega). To amplify the pWTY27 *repA* from the soil DNA, nested PCR amplifications were employed [42]. The first round of a PCR reaction was performed using primers (5^′^-CAGGTCAGGGTGCCCATGCCGTAC-3^′^, 5^′^-CGTACCCGCCTTGTACGTCCGCAG-3^′^) and KOD FX enzyme (Toyoba) under conditions (98°C 10 s, 60°C 30 s, 68°C 40 s for 30 cycles), and then 1 μl PCR product was added for the second round of the PCR reaction with primers (5^′^-CGGTCGCTCTGCTGCACCCAG-3^′^, 5^′^-GCGAGCCCAAGATTCACCGTCTG-3^′^) under conditions (98°C 10 s, 58°C 30 s, 68°C 30 s for 20 cycles). Similarly, to amplify the Y27 *oriC*, two primers (5^′^-ATGCACGCCGACCGCAAGATC-3^′^, 5^′^-AYRSGTTGCCGAACAGTGGACA-3^′^) were used for the first round, and nested primers (5^′^-CCACGGCCCCGAATCCGCCTC-3^′^, 5^′^- GCACAACACCGGCCTGCCTGTG-3^′^) for the second round of the PCR reactions. To amplify the A3(2) *oriC*, primers used in the first round reaction were the same as in the Y27 *oriC*, and new nested primers (5^′^-GCCTTTCCCATGCCCCT.GGGT-3^′^, 5^′^-CCTGCCCTGATGATCCCTCACCAG −3^′^) for the second round of the PCR reactions.

## Competing interest

The authors declare no conflict of interest.

## Authors’ contributions

TW designed and performed all the experiments. ZC, QC, MZ, XT and LZ isolated endophytic *Streptomyces* strains and identified plasmids. PX and MS constructed plasmids. ZJQ was involved in project design, and prepared the manuscript. All authors read and approved the final manuscript.

## Supplementary Material

Additional file 1 **Figure S1.** Identification of fourteen indigenous plasmids. Fourteen plasmids from endophytic Streptomyces strains were digested with NcoI and electrophoresed in 1% agarose gel at 6.7 V/cm for 4 h. Sizes of five bands are indicated. Click here for file

Additional file 2 **Figure S2.** Features of the 1136-bp sequence of the Y27 chromosomal oriC between the dnaA and dnaN genes. Taking the conserved DnaA binding-boxes of 9 bp (TTGTCCACA) in the S. lividans oriC as a reference [24], 25 DnaA binding-boxes of 9 bp (forward indicated by arrowheads and reverse by dashed arrowheads) for the Y27 oriC are predicted by the Vector NTI® 9.0 software (Invitrogen). Two AT-rich sequences are boxed. Click here for file

Additional file 3 **Figure S3.** Identification of fourteen endophytic Streptomyces strains. The plug-embedded mycelium of fourteen endophytic Streptomyces strains was digested with SspI and electrophoresed in a 1.0% pulsed-field gel at 8.6 V/cm, 10 s to 60 s switch time and 14oC for 22 h. Click here for file

Additional file 4 **Figure S4.** Schematic map of pWTY27. Predicted ORFs and their transcription directions are indicated by arrowheads. The replication (repA and repB), transfer (traA) and other genes (int: integrase; phc: phage capsid; kor: kill-override; spd: spread) and site (iteron) are shown. (JPEG 32 kb) Click here for file

Additional file 5 **Table S1.** Predicted ORFs of plasmid pWTY27. Detailed information and possible functions of the fifteen ORFs of pWTY27. Click here for file
