# Supplementary figures and images for: Characterization of replication and conjugation of plasmid pWTY27 from a widely distributed Streptomyces species
Source: BMC Microbiol. 2012 Nov 7;12:253. doi: 10.1186/1471-2180-12-253 (PMC3583192; doi:10.1186/1471-2180-12-253)

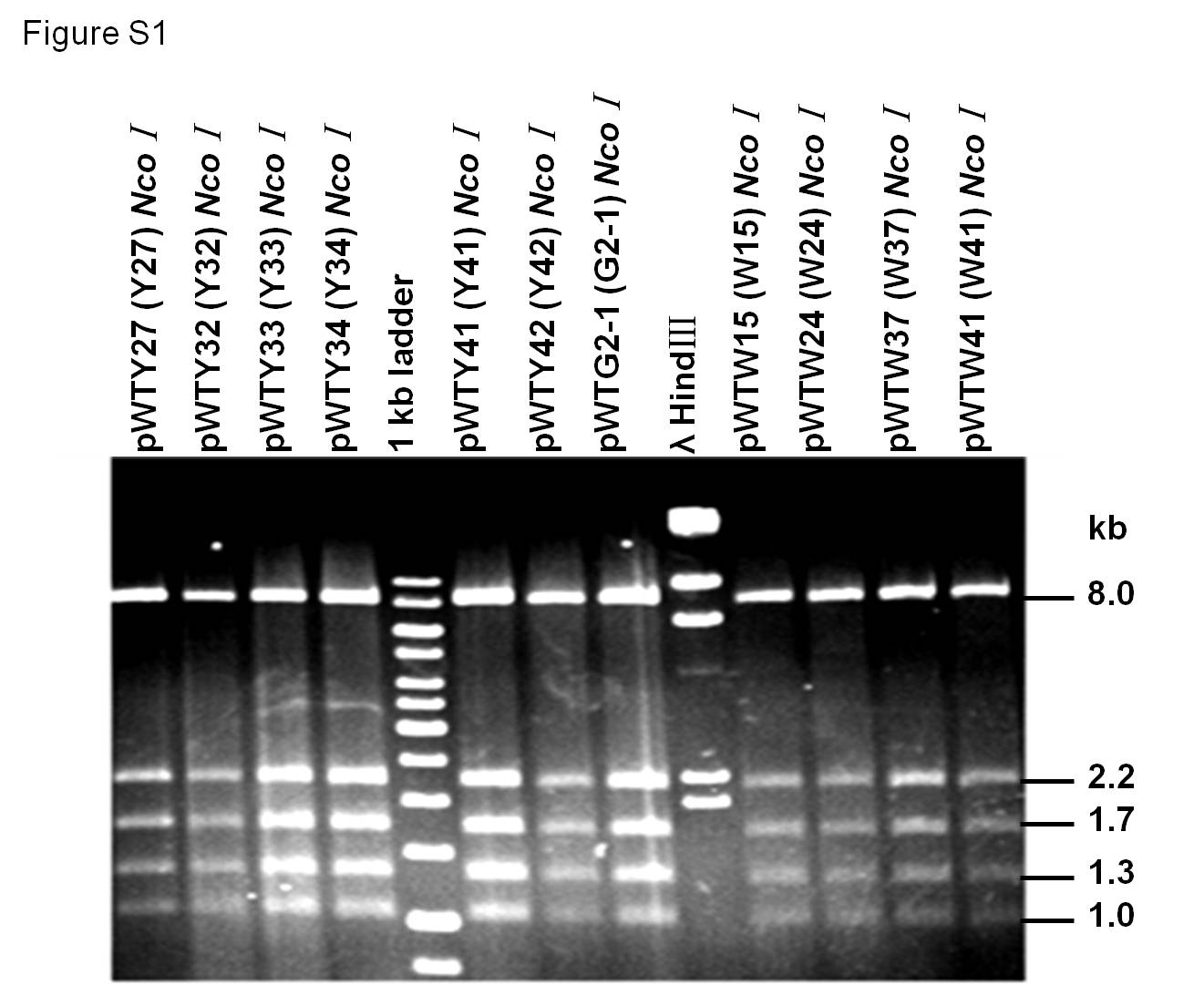

Supplement: Additional file 1 — Figure S1. Identification of fourteen indigenous plasmids. Fourteen plasmids from endophytic Streptomyces strains were digested with NcoI and electrophoresed in 1% agarose gel at 6.7 V/cm for 4 h. Sizes of five bands are indicated. [file 1471-2180-12-253-S1.jpeg]

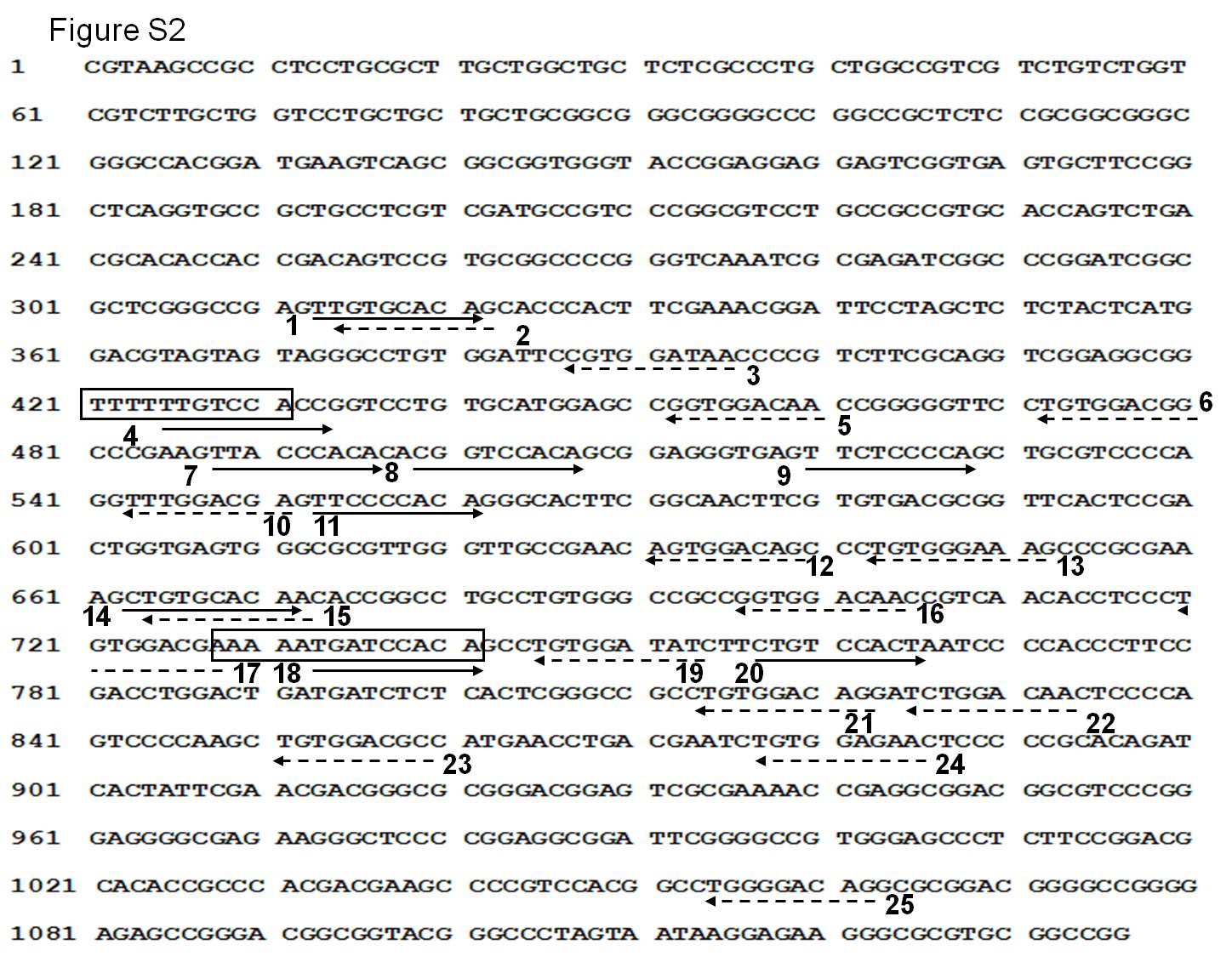

Supplement: Additional file 2 — Figure S2. Features of the 1136-bp sequence of the Y27 chromosomal oriC between the dnaA and dnaN genes. Taking the conserved DnaA binding-boxes of 9 bp (TTGTCCACA) in the S. lividans oriC as a reference [24], 25 DnaA binding-boxes of 9 bp (forward indicated by arrowheads and reverse by dashed arrowheads) for the Y27 oriC are predicted by the Vector NTI® 9.0 software (Invitrogen). Two AT-rich sequences are boxed. [file 1471-2180-12-253-S2.jpeg]

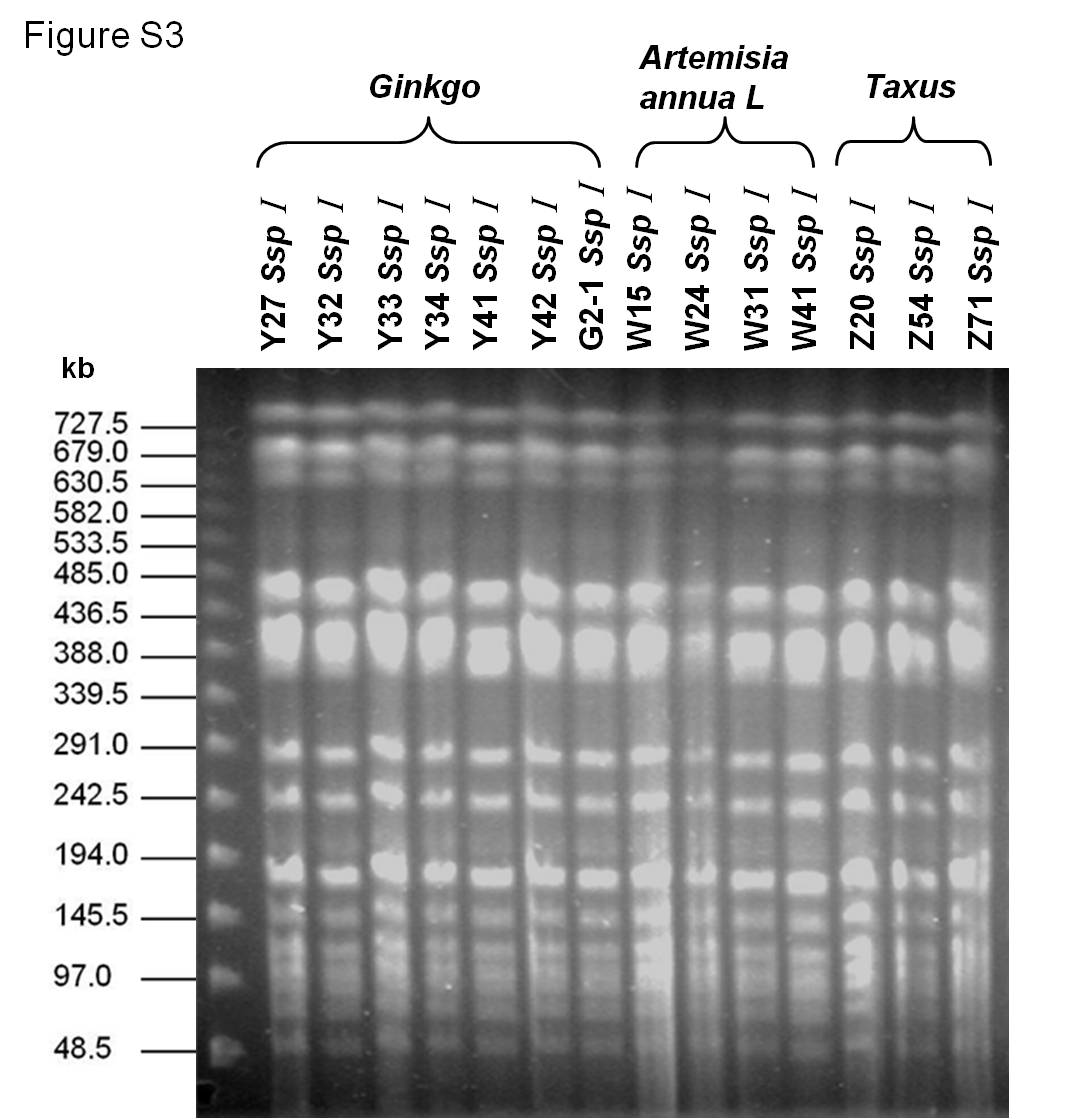

Supplement: Additional file 3 — Figure S3. Identification of fourteen endophytic Streptomyces strains. The plug-embedded mycelium of fourteen endophytic Streptomyces strains was digested with SspI and electrophoresed in a 1.0% pulsed-field gel at 8.6 V/cm, 10 s to 60 s switch time and 14oC for 22 h. [file 1471-2180-12-253-S3.jpeg]

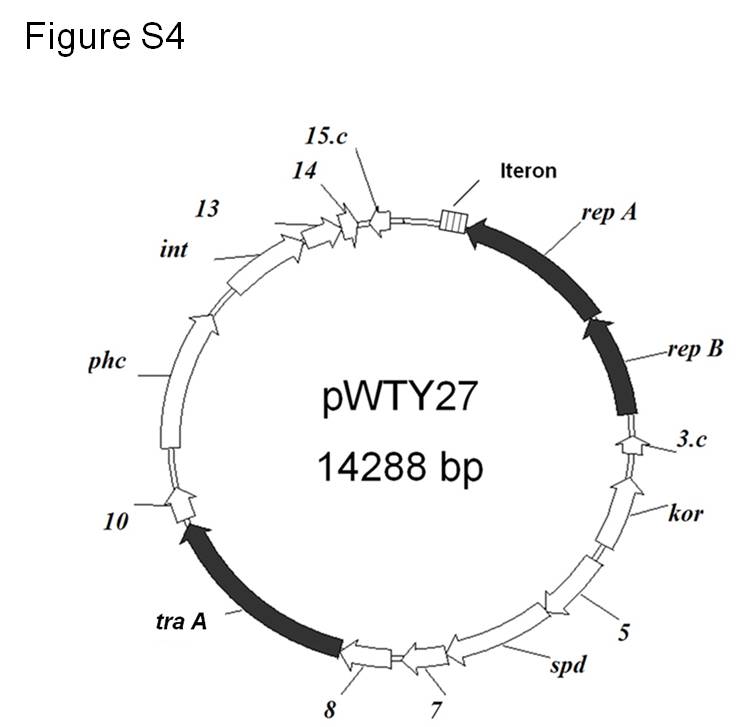

Supplement: Additional file 4 — Figure S4. Schematic map of pWTY27. Predicted ORFs and their transcription directions are indicated by arrowheads. The replication (repA and repB), transfer (traA) and other genes (int: integrase; phc: phage capsid; kor: kill-override; spd: spread) and site (iteron) are shown. (JPEG 32 kb) [file 1471-2180-12-253-S4.jpeg]
