# Supplementary material for: Characterization of replication and conjugation of plasmid pWTY27 from a widely distributed Streptomyces species
Source: BMC Microbiol. 2012 Nov 7;12:253. doi: 10.1186/1471-2180-12-253 (PMC3583192; doi:10.1186/1471-2180-12-253)
Supplement: Additional file 5 — Table S1. Predicted ORFs of plasmid pWTY27. Detailed information and possible functions of the fifteen ORFs of pWTY27. [file 1471-2180-12-253-S5.doc]

**TABLE S1** Predicted ORFs of the 14288 bp sequence of plasmid pWTY27

| ORFs | Position (bp) | Size (aa) | | E. value | Closest resemblance (organisms) |
| --- | --- | --- | --- | --- | --- |
| pWTY27.1c  pWTY27.2c  pWTY27.3c  pWTY27.4c  pWTY27.5  pWTY27.6  pWTY27.7  pWTY27.8  pWTY27.9  pWTY27.10  pWTY27.11  pWTY27.12  pWTY27.13  pWTY27.14  pWTY27.15c | 2189–621  3283–2195  3699–3502  4697–3939  4881–5600  5597–6694  6691–7140  7237–7755  7752–9818  9870-10220  10627–12102  12436–13281  13304–13690  13687–13851  14173–13958 | 522  362  65  252  239  365  149  172  688  116  491  281  128  54  71 | 4 × 10^-43^  2 × 10^-94^  2 × 10^-13^  2 × 10^-8^  5 × 10^-15^  2 × 10^-55^  1 × 10^-27^  1 × 10^-26^  0  7 × 10^-55^  3 × 10^-121^  2 × 10^-13^ | | Hypothetical protein StrviDRAFT_6922 (*Streptomyces violaceusniger*)  DNA primase/polymerase of pSG2 (*Streptomyces ghanaensis*)  Hypothetical protein SSFG_07858 (*Streptomyces ghanaensis*)  TraR of pSVH1 (*Streptomyces venezuelae*)  Hypothetical protein SACT1_3833 (*Streptomyces griseus* XylebKG-1.)  SpdB2 of pSG2 (*Streptomyces ghanaensis*)  Hypothetical protein STSU_33580 (*Streptomyces tsukubaensis* NRRL18488*r*)  TraA of pSG2 (*Streptomyces ghanaensis*)  TraB of pSG2 (*Streptomyces ghanaensis*)  Unknown  Phage head capsid protein (*Nocardia farcinica*)  Resolvase of pSG2 (*Streptomyces ghanaensis*)  Hypothetical protein SSFG_07855 (*Streptomyces ghanaensis*)  Unknown  Unknown |
